# Association of cancer treatment with excess heart age among five-year young breast cancer survivors

**DOI:** 10.1007/s11764-024-01645-9

**Published:** 2024-07-15

**Authors:** Jacqueline B. Vo, Shoshana Rosenberg, Bessie X. Zhang, Craig Snow, Greg Kirkner, Philip D. Poorvu, Rachel Gaither, Kathryn J. Ruddy, Rulla M. Tamimi, Jeffrey M. Peppercorn, Lidia Schapira, Virginia F. Borges, Steven E. Come, Anju Nohria, Ann H. Partridge

**Affiliations:** 1 Cancer Prevention Fellowship Program, Division of Cancer Epidemiology & Genetics, National Cancer Institute, 9609 Medical Center Drive, 7E532, Bethesda, Rockville, MD 20906 USA; 2 Department of Medical Oncology, Dana-Farber Cancer Institute, Boston, MA USA; 3 Department of Population Health Sciences, Weill Cornell Medicine, New York, NY USA; 4 Harvard Medical School, Boston, MA USA; 5 Department of Oncology, Mayo Clinic, Rochester, MN USA; 6 Department of Epidemiology, Brown University School of Public Health, Providence, RI USA; 7 Department of Medicine, Massachusetts General Hospital, Boston, MA USA; 8 Department of Medicine, Stanford Cancer Institute, Palo Alto, CA USA; 9 Division of Medical Oncology, University of Colorado Cancer Center, Aurora, CO USA; 10 Department of Medicine, Breast Oncology Program, Beth Israel Deaconess Medical Center, Boston, MA USA; 11 Cardiovascular Division, Brigham and Women’s Hospital, Boston, MA USA

**Keywords:** Breast cancer survivorship, Young breast cancer, Cardiovascular disease risk, Excess heart age

## Abstract

**Purpose:**

Data evaluating cardiovascular disease (CVD) risk by cancer treatment among young women (≤ 40 years) with breast cancer are limited.

**Methods:**

Among 372 five-year breast cancer survivors aged 30–40 years from the Young Women's Breast Cancer Study, we assessed the association of cancer treatments (anthracyclines, trastuzumab, radiation/laterality, endocrine therapy) and excess heart age (difference between predicted 10-year CVD risk as assessed by adapted Framingham Risk Score and chronological age), prevalent elevated excess heart age (≥ 2 years), and worsening excess heart age (change of ≥ 2 excess heart age years) at breast cancer diagnosis and two- and five-year follow-up using multivariable linear and logistic regressions.

**Results:**

Most women had stage I or II (79%), ER + (71%), or PR + (65%) breast cancer. At diagnosis, women had little excess heart age by treatment receipt (range of means = -0.52,0.91 years). Left-sided radiation (β = 2.49,SE = 0.96,p = 0.01) was associated with higher excess heart age at five-year follow-up. For prevalent elevated excess heart age (two-year = 26%;five-year = 27%), women treated with right-sided radiation had increased risk at two-years (OR = 2.17,95%CI = 1.12–4.19), yet at five-years, associations were observed after any radiation (OR = 1.92,95%CI = 1.09–3.41), especially after left-sided (OR = 2.13,95%CI = 1.09–3.41) radiation. No associations were observed between systemic treatments and prevalent elevated excess heart age or any treatments with worsening excess heart age.

**Conclusions:**

Among young breast cancer survivors, radiation, but not other cancer treatments, was associated with elevated excess heart age.

**Implications for cancer survivors:**

CVD risk tools that incorporate cancer treatment, such as radiation, are needed to identify high risk young breast cancer survivors given the long survivorship and long latency of cardiovascular disease.

**Supplementary Information:**

The online version contains supplementary material available at 10.1007/s11764-024-01645-9.

## Introduction

With improved survival due to advances in treatment [1], young adults with breast cancer (diagnosed at 40 years of age or younger) are likely to have long periods of survivorship and, thus, may prematurely develop cardiovascular disease resulting from late effects of cancer treatment. Young patients are often diagnosed with more advanced disease and may need more aggressive cancer treatments [2]. Cancer treatment such as anthracyclines, trastuzumab, radiation, and endocrine therapy are associated with cardiotoxicity [3–7]. Data are limited surrounding cardiovascular disease risk for young breast cancer survivors; however, there is extensive research in childhood cancer survivors and adolescent and young adult (AYA) cancer survivors who receive similar treatments that have characterized late cardiotoxicity after cancer treatment [8–11]. For example, childhood cancer survivors have a 15 times higher risk of developing heart failure and 10 times higher risk of developing coronary artery disease relative to their siblings [9], and AYA cancer survivors (including those with breast cancer) have a two-fold increased risk of developing any cardiovascular disease compared to individuals without cancer [10]. Further, the risk of mortality among AYA cancer survivors who develop cardiovascular disease is 11-fold higher compared to cancer survivors without cardiovascular disease [10]. Mechanisms by which cancer treatments affect the cardiovascular disease system in AYA cancer survivors could be similar, yet clinical relevance to young breast cancer survivors may be limited due to differences in cancer types and treatments, gender, and age.

Current clinical guidelines in the U.S. provide recommendations for screening and monitoring of cardiovascular disease in patients receiving cardiotoxic cancer treatments [12–14]. However, recommendations are limited to 1–2 years post breast cancer diagnosis, and younger patients who receive cardiotoxic treatments may be at increased risk for developing cardiovascular disease many years or even decades later. A prior study conducted among young breast cancer survivors demonstrated that cardiovascular disease risk, measured using excess heart age, increased two years after diagnosis among those receiving endocrine therapy [15]. To expand on these findings, the present study was conducted in a larger, multicenter prospective cohort of women diagnosed with breast cancer at age 40 years or younger, and examined associations between cancer treatment and excess heart age at two and five years following diagnosis. Because cardiovascular disease is the leading cause of non-cancer deaths among breast cancer survivors [16, 17], evaluating cardiovascular risk among the youngest survivors, where data are limited, can inform cardiovascular disease prevention and monitoring in long-term follow-up.

## Methods

### Study cohort

The Young Women’s Breast Cancer Study (YWS) enrolled 1,302 women aged ≤ 40 years and diagnosed with breast cancer between 2006–2016 across 13 academic and community‐based centers in the United States and Canada. YWS was approved by the Institutional Review Board at the Dana‐Farber/Harvard Cancer Center and other participating sites. Eligible participants were identified through rapid case ascertainment or clinic list review within 6 months of their breast cancer diagnosis, and an invitation letter was sent to women to participate. Data from participants included serial surveys (collected every 6 months in the first 3 years and annually thereafter), and clinical data were abstracted from medical record review. Supplemental medical record abstraction was conducted for the present study to obtain blood pressures which were not collected as part of the standard medical record abstraction.

### Analytic population

Inclusion criteria for the present analysis were women diagnosed with Stage 0-III breast cancer between the ages 30–40 years, who had follow-up data for at least five years (defined as women who completed either the four-, five-, or six-year survey). We initially excluded women who completed abbreviated surveys (*n* = 91) or did not have accessible follow-up data in electronic medical records (*n* = 247). Among patients with available survey and medical record data, we further excluded individuals who had a self-reported a history of cardiovascular disease on the baseline survey (*n* = 1), were pregnant at the time of survey administration (*n* = 24), had stage IV disease at diagnosis or who developed a distant or local recurrence of breast cancer within the first 5 years of diagnosis (*n* = 19), and those missing any heart age variables (i.e., blood pressure, blood pressure medication use, body mass index, history of diabetes, and smoking status) (*n* = 70). **Supplemental **Fig. 1 demonstrates a consort diagram for selection of the study cohort.

### Study outcome

Our primary outcome was **excess heart age** calculated at two- and five-years post-diagnosis. Heart age is an adapted version of the Framingham Risk Score, which is a 10-year estimate of cardiovascular disease risk [18] and is a gender-based equation calculated using chronological age (continuous), systolic blood pressure (defined for this analysis as closest blood pressure within 6 months of diagnosis date and two- and five-year post diagnosis), antihypertensive medication use (yes/no), body mass index (continuous), history of smoking within the last year (yes/no), and history of diabetes (yes/no). The upper limit of heart age was set at 100. Excess heart age is equal to the difference between heart age and chronological age and represents the excess risk for cardiovascular events [18].

Second, we examined **prevalent elevated excess heart age** (an indicator of poorer cardiovascular disease health) defined as an excess heart age ≥ two years at both follow-up timepoints. Third, to examine factors that were associated with a **worsening excess heart age**, we examined change in excess heart age from baseline (time closest to breast cancer diagnosis and prior to treatment receipt) to follow-up [excess heart age at follow-up – excess heart age at baseline]. Worsening excess heart age was defined as an increase of at least two heart age years, and patients could have “healthy cardiovascular disease risk” (excess heart age less than 0) and become “less healthy” if the change was at least 2 excess heart age years. Among the analytic population of five-year breast cancer survivors, 24 women did not have survey data at two-year follow-up.

### Cancer treatments

We examined the following cancer treatments: anthracyclines (yes, no), trastuzumab (yes, no), radiation (yes, no), and endocrine therapy (yes, no, missing). We further assessed radiation by laterality (left, right) due to the potential increased risk of cardiovascular disease after left-sided radiation [5, 19]. Systemic endocrine therapy (including tamoxifen and aromatase inhibitors; yes, no, missing) was determined by use at the 18 months post diagnosis.

### Statistical analyses

Descriptive statistics (mean and standard deviation, frequency and percentages) were used to characterize the study population. Paired-sample t-tests and Mcnemar’s chi-square statistics were used to assess differences in excess heart age between baseline and follow-up at two- and five-years.

To determine whether there was an association between cancer treatment and excess heart age (continuous) at two-year follow-up and five-year follow-up, we conducted multivariable linear regression. Further, to assess the association between cancer treatment and 1) prevalent elevated excess heart age (dichotomized) at two-year and five-year follow-up and 2) worsening excess heart age (dichotomized) between baseline and two-year follow-up, between baseline and five-year follow-up, and between two-year and five-year follow-up, we conducted multivariable logistic regression. All models adjusted for age at diagnosis (continuous), race (White versus all other races due to small sample sizes), stage (0, I, II, III), and other cancer treatments (anthracycline, trastuzumab, any radiation, radiation and laterality, or endocrine therapy). All tests were two-sided, and significance was set at *p* < 0.05. STATA version 17.0 was used.

## Results

### Study population characteristics

**Fig. 1:**
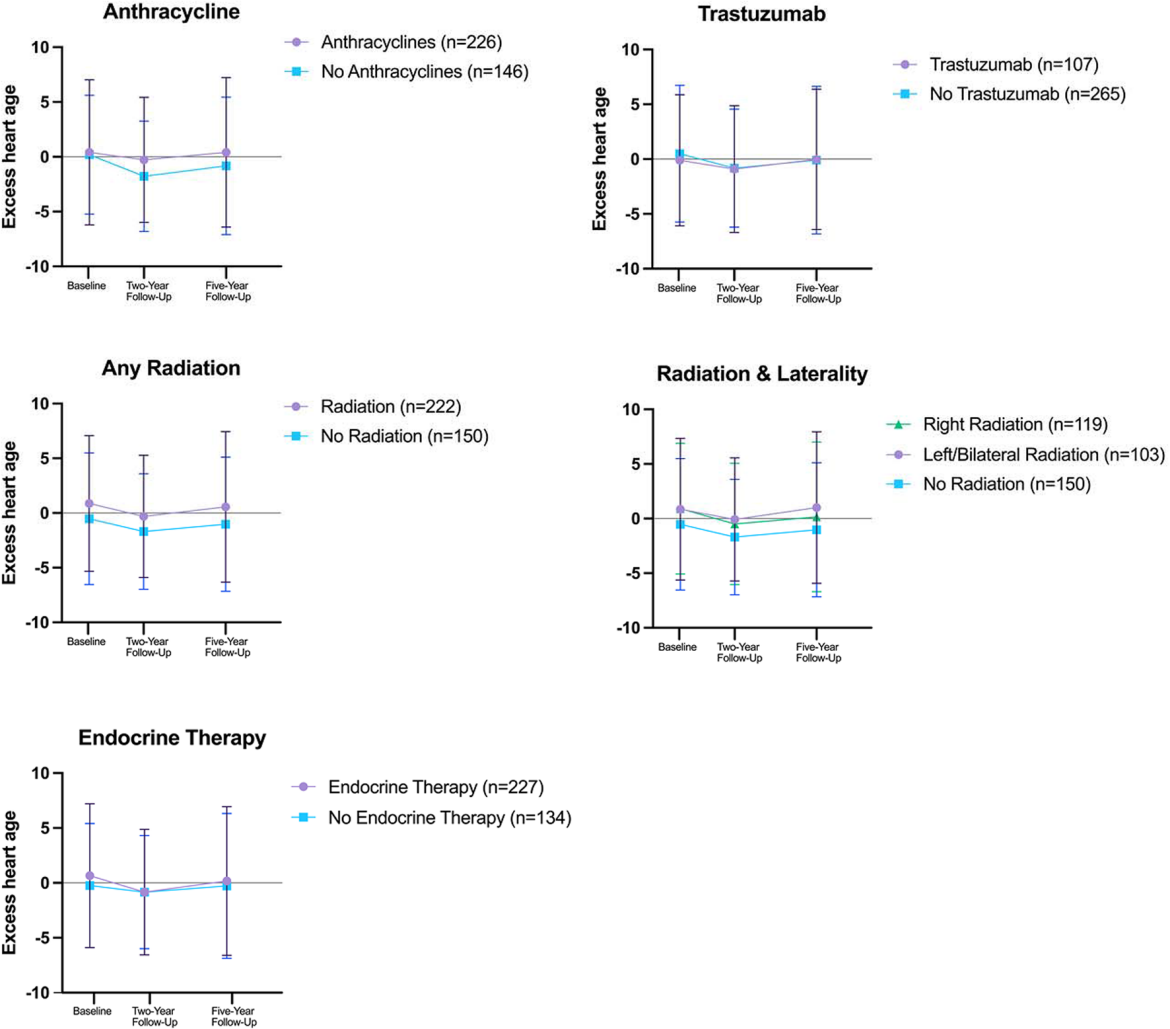
Excess heart age over the follow-up period, by cancer treatment type. Note. Treatment types are not mutually exclusive

Among 1,302 YWS participants, 372 met inclusion criteria and had survived at least five years after breast cancer diagnosis (Fig. 1). The median age at breast cancer diagnosis was 37.5 years (interquartile range: 35.4–39.6 years) (Table 1). Participants were 93% White, 97% non-Hispanic/Latina, 85% college graduates, 80% married or with a partner, and 68% employed at the time of breast cancer diagnosis. Most patients had either Stage I (40%) or Stage II (39%), and Hormone Receptor [HR] + /HER2-(47%) or HR + /HER2 + breast (21%) cancers. Most (73%) women received chemotherapy, including 40% who received chemotherapy regimens with anthracyclines and no trastuzumab, 8% who received trastuzumab and no anthracyclines, 20% who received both anthracyclines and trastuzumab, and 4% who received other chemotherapy. Nearly 60% of women received radiation, and all women received surgery (30% had breast conserving surgery, 25% unilateral mastectomy, and 45% bilateral mastectomy). None of the 372 women were diagnosed with clinically evident cardiovascular disease after five years of follow-up.

**Table 1:** Patient and clinical characteristics of 372 women in the Young Women's Breast Cancer Study

Characteristic	*N* = 372 (100.00%)
Age at breast cancer diagnosis, median (IQR)	37.5 (35.4–39.6)
Stage, *n* (%)	
0	34 (9.14%)
I	149 (40.05%)
II	146 (39.25%)
III	43 (11.56%)
Breast subtype^a^, *n* (%)	
HR + /HER2 +	79 (21.24%)
HR-/HER2 +	39 (10.48%)
HR + /HER2-	173 (46.51%)
HR-/HER2-	58 (15.59%)
Unknown subtype	23 (6.18%)
Laterality, *n* (%)	
Bilateral	4 (1.08%)
Left	176 (47.31%)
Right	192 (51.61%)
Menopausal status at breast cancer diagnosis, *n* (%)	
No	148 (39.78%)
Yes	118 (31.72%)
Missing	106 (28.49%)
Race, *n* (%)	
American Indian or Alaskan Native	1 (< 1%)
Asian	14 (3.76%)
Black	5 (1.34%)
White	347 (93.28%)
Other/Unknown/Multi-Racial	5 (1.34%)
Ethnicity, *n* (%)	
Hispanic/Latina	13 (3.49%)
Non-Hispanic/Latina	359 (96.51%)
Education level, *n* (%)	
No college degree	54 (14.52%)
College degree	318 (85.48%)
Marital status, *n* (%)	
Not married/partner	74 (19.89%)
Married/partner	297 (79.84%)
Missing	1 (0.27%)
Employment status, *n* (%)	
Not employed	119 (31.99%)
Employed full-time or part-time	253 (68.01%)
Chemotherapy, *n* (%)	
Any anthracyclines	226 (60.75%)
Any trastuzumab	107 (28.76%)
Anthracyclines and trastuzumab	76 (20.43%)
Anthracyclines, no trastuzumab	150 (40.32%)
Trastuzumab, no anthracyclines	31 (8.33%)
Other chemotherapy	16 (4.30%)
No chemotherapy	99 (26.61%)
Radiation, *n* (%)	
None	150 (40.32%)
Yes	222 (59.68%)
Radiation and laterality, *n* (%)	
Right radiation	119 (31.99%)
Left or bilateral radiation	103 (27.69%)
No radiation	150 (40.32%)
Surgery, *n* (%)	
Bilateral mastectomy	169 (45.43%)
Lumpectomy	110 (29.57%)
Unilateral mastectomy	93 (25.00%)
Radiation and surgery, *n* (%)	
Radiation and lumpectomy	110 (29.57%)
Radiation and bilateral mastectomy	61 (16.40%)
Radiation and unilateral mastectomy	51 (13.71%)
No radiation	150 (40.32%)
Endocrine therapy at 18 months, *n* (%)	
No	134 (36.02%)
Yes	227 (61.02%)
Unknown	11 (2.96%)

Acronyms: HR = hormone receptor. HER2 = Human epidermal growth factor receptor 2. ^a^Following the Surveillance, Epidemiology, and End Results manual, Hormone Receptor was categorized as positive if estrogen receptor status or progesterone receptor status were positive. Hormone Receptor was categorized as negative if estrogen receptor status and progesterone receptor status were both negative

### Excess heart age

At baseline, women who went on to receive anthracyclines had slightly higher excess heart age than those who did not (0.40 vs 0.19 years, respectively). The same was observed among those who received radiation versus not (0.88 vs -0.52 years, respectively) (Table 2). Women who went on to receive trastuzumab had a lower excess heart age than those who did not (-0.11 vs 0.49 years, respectively). Over time, the difference in excess heart age between women who received anthracyclines and those who did not increased slightly at two- and five-year follow-ups, whereas the difference between women who received radiation versus those who did not was consistent over time (Fig. 1). Women who received trastuzumab or endocrine therapy had similar excess heart age at follow-up, compared to those who did not receive trastuzumab or endocrine therapy. At two-year follow-up, none of the cancer treatments were significantly associated with increasing excess heart age in the multivariable models (Table 2). However, at five-year follow-up, radiation treatment was associated with increasing excess heart age (β = 1.92, SE = 0.82, *p* = 0.02), which was largely driven by left-sided radiation (β = 2.49, SE = 0.96, *p* = 0.01). At five-year follow-up, no associations were observed for anthracyclines, trastuzumab, or endocrine therapy.

**Table 2:** Excess heart age at breast cancer diagnosis, two-year follow-up, and five-year follow-up by cancer treatment type

	Baseline excess heart age (*n* = 372)	Two-year follow-up excess heart age (*n* = 348)	Two-year follow-up excess heart age			Five-year follow-up excess heart age (*n* = 372)	Five-year follow-up excess heart age		
Variable	Years, mean (SD)	Years, mean (SD)	β	SE	*p*-value	Years, mean (SD)	β	SE	*p*-value
Anthracyclines									
Yes	0.40 (6.62)	− 0.28 (5.71)	1.52	0.77	0.051	0.40 (6.82)	1.20	0.91	0.19
No	0.19 (5.41)	− 1.78 (5.04)	REF	−	−	− 0.83 (6.27)	REF	−	−
Trastuzumab									
Yes	− 0.11 (5.99)	− 0.91 (5.78)	− 0.16	0.67	0.81	− 0.03 (6.40)	− 0.05	0.78	0.95
No	0.49 (6.24)	− 0.83 (5.39)	REF	−	−	− 0.10 (6.74)	REF	−	−
Any radiation									
Yes	0.88 (6.21)	− 0.31 (5.59)	1.36	0.71	0.056	0.56 (6.89)	**1.92**	**0.82**	**0.02**
No	− 0.52 (6.01)	− 1.69 (5.29)	REF	−	−	− 1.03 (6.14)	REF	−	−
Radiation and laterality									
Left or bilateral	0.85 (6.49)	− 0.08 (5.64)	1.53	0.83	0.07	1.01 (6.93)	**2.49**	**0.96**	**0.01**
Right	0.91 (5.99)	− 0.50 (5.56)	1.23	0.78	0.12	0.16 (6.85)	1.48	0.90	0.10
No radiation	− 0.52 (6.01)	− 1.69 (5.29)	REF	−	−	− 1.03 (6.14)	REF	−	−
Endocrine therapy									
Yes	0.66 (6.55)	− 0.84 (5.72)	0.04	0.66	0.96	0.17 (6.77)	0.04	0.77	0.96
No	− 0.25 (5.66)	− 0.85 (5.15)	REF	−	−	− 0.28 (6.60)	REF	−	−
Unknown	0.29 (2.85)	DS	DS			− 2.77 (3.06)	− 2.81	2.09	0.18

β = beta coefficient, SE = standard error, REF = reference group, DS = data suppressed due to *n* < 10. Linear regression models adjusted for age at cancer diagnosis (continuous), race (white/all other races), and cancer stage (0/I/II/III) and other cancer treatments when appropriate: anthracyclines (yes/no), trastuzumab (yes/no), radiation (yes/no), endocrine therapy (yes/no/missing). Bold indicative of statistical significance

### Prevalent elevated excess heart age

At two years, 26% (*n* = 90/348) of young breast cancer survivors had prevalent excess heart age of ≥ 2 years (Table 3). There were no significant associations between the various cancer treatments and prevalent excess heart age at two-years, except for right-sided radiation which had a two-fold increased risk (OR = 2.17, 95%CI = 1.12–4.19). At five years, 27% (*n* = 100/372) of young breast cancer survivors had a prevalent excess heart age of ≥ 2 years. Any radiation was associated with an increased odds of prevalent elevated excess heart age (OR = 1.92, 95%CI = 1.09–3.41), with left-sided radiotherapy associated with higher odds (OR = 2.13, 95%CI = 1.09–3.41) but the association was attenuated for right-sided radiotherapy (OR = 1.78, 95%CI = 0.94–3.33).

**Table 3:** Association of prevalent elevated excess heart age by cancer treatment type, at two- and five-year follow-up

	Two-year follow-up Prevalent elevated excess heart age (*N* = 348)			Five-year follow-up Prevalent elevated excess heart age (*N* = 372)		
Cancer treatment	*N*	OR	95% CI	*N*	OR	95% CI
Anthracyclines						
Yes	65	1.54	(0.79 − 2.98)	62	1.19	(0.64 − 2.22)
No	25	REF	−	38	REF	−
Trastuzumab						
Yes	26	0.86	(0.49 − 1.49)	27	0.89	(0.52 − 1.53)
No	64	REF	−	73	REF	−
Radiation						
Yes	65	1.84	(0.99 − 3.89)	66	**1.92**	**(1.09 − 3.41)**
No	25	REF	−	34	REF	−
Radiation and laterality						
Left or bilateral	26	1.46	(0.71 − 2.98)	32	**2.13**	**(1.10 − 4.14)**
Right	39	**2.17**	**(1.12 − 4.19)**	34	1.78	(0.94 − 3.33)
No radiation	25	REF	−	34	REF	−
Endocrine therapy						
Yes	58	0.98	(0.57 − 1.68)	65	1.03	(0.61 − 1.74)
No	32	REF	−	35	REF	−
Unknown	0	DS		0	DS	

OR = Odds Ratio, CI = confidence intervals, REF = reference group, DS = data suppressed due to *n* < 10. Models adjusted for age at cancer diagnosis (continuous), race (white/all other races), and cancer stage (0/I/II/III) and other cancer treatments when appropriate: anthracyclines (yes/no), trastuzumab (yes/no), radiation (yes/no), endocrine therapy (yes/no/missing). Bold indicative of statistical significance. Prevalent elevated excess heart age defined as excess heart age ≥ 2 years

### Worsening excess heart age (Increase of ≥ 2 excess heart age years)

Between baseline and two-year follow-up, 22% (*n* = 75/348) of women had an increase of at least 2 excess heart age years. Between baseline and five-year follow-up, 31% (*n* = 114/372) women had an increase of at least 2 excess heart age years. Between two-year follow-up and five-year follow-up, 40% (*n* = 139/372) of women had an increase of at least 2 excess heart age years. Across all three time points, there were no associations between cancer treatments and worsening excess heart age (**Supplemental **Table 1).

## Discussion

In this cohort of young breast cancer survivors, most women had minimal excess heart age at the time of breast cancer diagnosis, suggesting a cardiovascular disease risk comparable to the nationally reported average of excess heart age for women aged 30–39 years [20]. However, nearly 1/3 of young breast cancer survivors in this cohort experienced a change in their excess heart age from breast cancer diagnosis of ≥ 2 years after 5 years of follow-up. Among cancer treatments, radiation, especially left-sided radiation, was associated with higher cardiovascular disease risk after five-years of follow up, while other cancer treatments were not.

While studies have evaluated excess heart age in other patient populations[21–25], only one study has previously assessed excess heart age among breast cancer survivors. This prior study was conducted within an Alabama health system, and the mean excess heart age was 4.2 years at baseline for 152 women under 45 years [15]. The YWS cohort includes mostly non-Hispanic White patients largely based in Massachusetts, who have a statewide reported average excess heart age much lower than Alabama (3.5 vs 8.1 years, respectively) [20]. These differences are likely attributable to regional differences in cardiovascular disease risk factors including body mass index, comorbidities, and physical activity.

We have extended prior research by assessing the association between breast cancer treatment and excess heart age among young five-year survivors in a relatively large prospective cohort (twice the sample size of the Alabama study). We observed higher cardiovascular disease risk at five years for women treated with radiotherapy after breast cancer diagnosis. Cardiovascular disease, especially ischemic heart disease, is a long-term effect of radiation therapy with disease incidence occurring 10 + years after initial treatment [5, 26], with prior studies demonstrating increased risk after left- compared to right-sided radiotherapy [5, 19]. Since none of the patients in our study experienced cardiovascular events at 5 years follow-up, excess heart age may be capturing changes in cardiovascular risk factors, such as increasing blood pressure or body mass index, that may place young female survivors at risk for developing premature radiation-related cardiovascular disease, especially given the accentuated risk due to younger age at radiation exposure [13, 19, 27]. Further, the increased prevalent excess heart age observed after right-sided radiotherapy in one of our models may be related to an indirect effect with obesity, as women who are more obese and have large breasts may be less likely to receive mastectomies due to cosmetic challenges with surgical reconstruction and may be more likely to have partial mastectomy with radiation [28]. These higher-risk women may also have other co-occurring diseases or pre-existing risk factors that could potentially increase their risk of developing cardiovascular disease. Importantly, modern radiation treatment practices for cardioprotection, including positioning to protect the heart such as breath-hold or prone positioning, increased precision using image guidance, and proton therapy as an alternative energy source, could potentially improve long-term radiation-related cardiovascular disease risk [29]; however, we did not have these data available.

In the present analysis, we did not observe associations between excess heart age and anthracyclines, trastuzumab, or endocrine therapy that were found in a prior study [15]. This was reassuring and may be related to increased awareness about potential adverse effects of chemotherapy and resultant healthy lifestyle changes through cancer treatment and survivorship that mitigate cardiovascular disease risk [30]. It is also possible that compared to older patients, young women treated with anthracyclines and trastuzumab may be more resilient against cardiotoxic damage due to the relative lack of baseline cardiovascular disease risk factors [3, 4, 31]. However, it is important to note that subclinical changes in cardiac function that may occur after anthracyclines or trastuzumab receipt, especially when used together, could not be assessed with this excess heart age tool, and risk of cardiovascular disease among young breast cancer survivors may be heightened long after the five years of follow-up in this study as seen in AYA cancer survivors 10 + years after diagnosis [10].

Although we did not observe significant associations between anthracyclines or trastuzumab and excess heart age, clinical guidelines have stressed the importance of monitoring for and preventing cardiomyopathy in patients treated with anthracyclines or trastuzumab since early detection can lead to improved outcomes. Current clinical guidelines for breast cancer survivors do not emphasize screening after radiotherapy [12, 13]; however, we observed significant associations between radiation and subsequent elevated excess heart age. Thus, the clinical implications of this study include cardiovascular surveillance after radiotherapy and management of modifiable lifestyle factors (e.g., physical activity, weight management, hypertension control) to improve cardiovascular disease risk after radiation treatment. Excess heart age could be used in survivorship care to improve provider-patient communication regarding cardiovascular disease risk [18] and could facilitate healthy lifestyle changes or referral for additional screening as well as early detection and intervention for subclinical cardiovascular disease in high-risk women.

Findings of this research should be interpreted in the context of certain limitations, including our inability to assess cardiovascular risk associated with specific chemotherapy regimens (i.e., joint effects of anthracyclines with trastuzumab), radiation dose, or endocrine therapy type (tamoxifen versus aromatase inhibitors) due to inadequate statistical power. Further, five years of follow-up may not be a sufficient length of follow-up for young breast cancer survivors who are relatively healthy at diagnosis and due to the long latency of treatment-related cardiovascular disease, warranting future extended assessments of cardiovascular disease risk (e.g., 10 or more years after breast cancer diagnosis). The tool excess heart age may not fully capture the full range of cardiovascular risk prior to cancer treatment receipt, and we were also unable to assess left ventricular function which may have characterized subclinical changes from diagnosis to after treatment. Finally, the use of heart age is not validated in cancer populations, and studies are needed to inform cardiovascular disease risk associated with potentially cardiotoxic treatments, especially in patients where traditional cardiovascular risk estimation tools are unavailable. Future research should consider the cardiotoxic impact of newer regimens such as immunotherapies and cyclin-dependent kinase 4 and 6 (CDK4/6) inhibitors and the potential effects of ovarian suppression on cardiovascular disease risk.

Nevertheless, our finding of potentially increased risk of cardiovascular disease for a substantially small group of young breast cancer survivors, especially after left-sided radiotherapy, warrants future investigation especially given the long survivorship and long latency of cardiovascular disease. Cardiovascular disease risk tools that incorporate cancer treatment predictors into models are needed to appropriately identify high-risk patients, especially among young survivors who have a low absolute, but higher relative risk than age-matched non-cancer controls. Extended follow-up of the YWS cohort as well as evaluation of this risk in other cohorts may further quantify cardiovascular disease risk and long-term cardiac outcomes in young breast cancer survivors.

## Supplementary Information

Below is the link to the electronic supplementary material.

Supplementary file1 (DOCX 38 KB)

## Data Availability

The data that support the findings of this study are from the Young Women’s Breast Cancer Study, but restrictions apply to the availability of these data and are not publicly available.
