# Supplementary material for: Association of cancer treatment with excess heart age among five-year young breast cancer survivors
Source: J Cancer Surviv. 2024 Jul 15;20(1):189–97. doi: 10.1007/s11764-024-01645-9 (PMC12906554; doi:10.1007/s11764-024-01645-9)
Supplement: Supplementary file 1 — Supplementary file1 (DOCX 38 KB) [file 11764_2024_1645_MOESM1_ESM.docx]

**Supplemental Figure 1.** Consort diagram for selection of analytic population.

Total enrolled in the Young Women’s Breast Cancer Study

N=1302

Found to be ineligible post enrollment: (N=5)

- Language barriers: (n=2)
- Not patient’s first diagnosis of breast cancer: (n=2)
- Withdrew consent: (n=1)

Final YWS Cohort

N=1297

Baseline or 4/5/6 year survey not completed: (N=523)

- Completed abbreviated survey: (n=91)
- Baseline survey not completed: (n=22)
- Withdrawn from surveys: (n=20)
- Died before timepoint reached: (n=76)
- Died within 4-6 year survey window: (n=16)
- Lost to follow-up/did not complete 4, 5, or 6 year survey: (n=228)
- 4/5/6 year timepoint not reached as of June 2019: (n=71)

N=774

Excluded for subset analyses (N=402):

- Medical records not accessible: (n=247)
- Age <30: (n=41)
- Stage IV or recurrence: (n=19)
- Pregnant: (n=24)
- Cardiovascular disease reported on baseline survey: (n=1)
- Missing heart age variables: (n=70)

Total analytic sample

N = 372

**Supplemental Table 1.** Association of cancer treatment with worsening excess heart age years from breast cancer diagnosis to two- and five-year follow-up.

|  | **Baseline to 2-Year follow-up**  **Worsening elevated excess heart age**  **(N=348)** | | | **Baseline to 5-year follow-up**  **Worsening elevated excess heart age**  **(N=372)** | | | **2-year follow-up to 5-year follow-up**  **Worsening elevated excess heart age**  **(N=348)** | | |
| --- | --- | --- | --- | --- | --- | --- | --- | --- | --- |
| **Cancer Treatment** | **N** | **OR** | **95% CI** | **N** | **OR** | **95% CI** | **N** | **OR** | **95% CI** |
| Anthracyclines |  |  |  |  |  |  |  |  |  |
| Yes | 55 | 1.60 | (0.78-3.28) | 73 | 1.37 | (0.75-2.48) | 86 | 1.01 | (0.57-1.77) |
| No | 20 | REF | - | 41 | REF | - | 53 | REF | - |
| Trastuzumab |  |  |  |  |  |  |  |  |  |
| Yes | 24 | 1.00 | (0.56-1.79) | 41 | 1.64 | (0.99-2.79) | 43 | 1.14 | (0.70-1.86) |
| No | 51 | REF | - | 73 | REF | - | 96 | REF | - |
| Radiation |  |  |  |  |  |  |  |  |  |
| Yes | 49 | 0.90 | (0.47-1.71) | 64 | 0.79 | (0.46-1.36) | 87 | 1.29 | (0.77-2.17) |
| No | 26 | REF | - | 50 | REF | - | 52 | REF | - |
| Radiation |  |  |  |  |  |  |  |  |  |
| Left/bilateral | 24 | 0.90 | (0.43-1.90) | 32 | 0.89 | (0.47-1.67) | 44 | 1.65 | (0.89-3.04) |
| Right | 25 | 0.90 | (0.44-1.82) | 32 | 0.73 | (0.40-1.33) | 43 | 1.07 | (0.60-1.90) |
| No | 26 | REF | - | 50 | REF | - | 52 | REF | - |
| Endocrine Therapy |  |  |  |  |  |  |  |  |  |
| Yes | 46 | 0.89 | (0.50-1.56) | 71 | 0.93 | (0.56-1.53) | 91 | 1.05 | (0.65-1.70) |
| No | 29 | REF | - | 42 | REF | - | 48 | REF | - |
| Unknown | 0 | DS |  | <10 | DS |  | 0 | DS |  |
| Note. OR=Odds Ratio, CI=confidence intervals, REF=Reference group, DS=data suppressed due to <10 events. Models adjusted for age at cancer diagnosis (continuous), race (White/all other races), and cancer stage (0/I/II/III) and other cancer treatments when appropriate: anthracyclines (yes/no), trastuzumab (yes/no), radiation (yes/no), endocrine therapy (yes/no/missing). Bold indicative of statistical significance. Prevalent elevated excess heart age defined as excess heart age ≥ 2 years. | | | | | | | | | |
